# Supplementary figures and images for: Computerized history-taking improves data quality for clinical decision-making—Comparison of EHR and computer-acquired history data in patients with chest pain
Source: PLoS One. 2021 Sep 27;16(9):e0257677. doi: 10.1371/journal.pone.0257677 (PMC8476015; doi:10.1371/journal.pone.0257677)

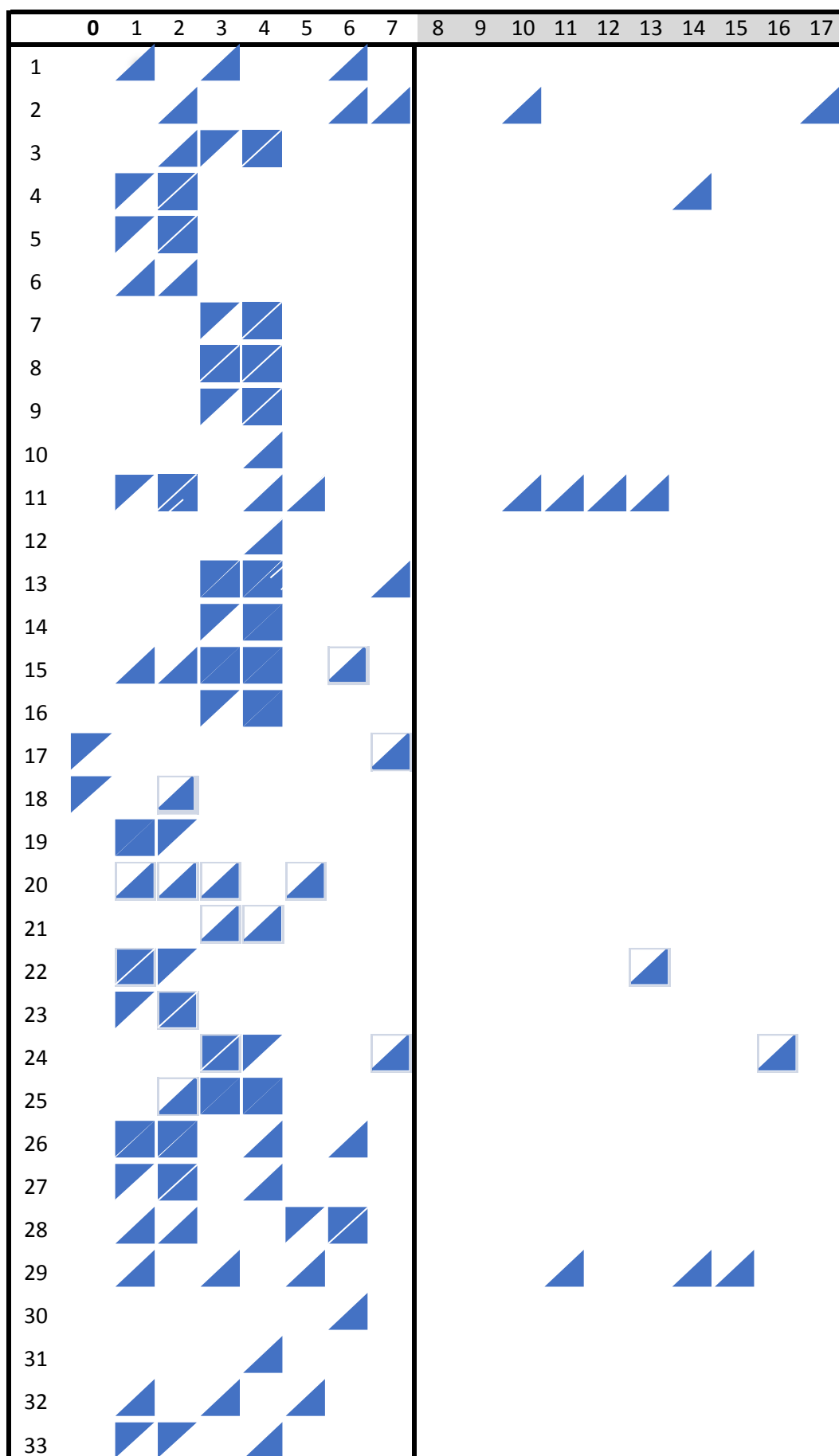



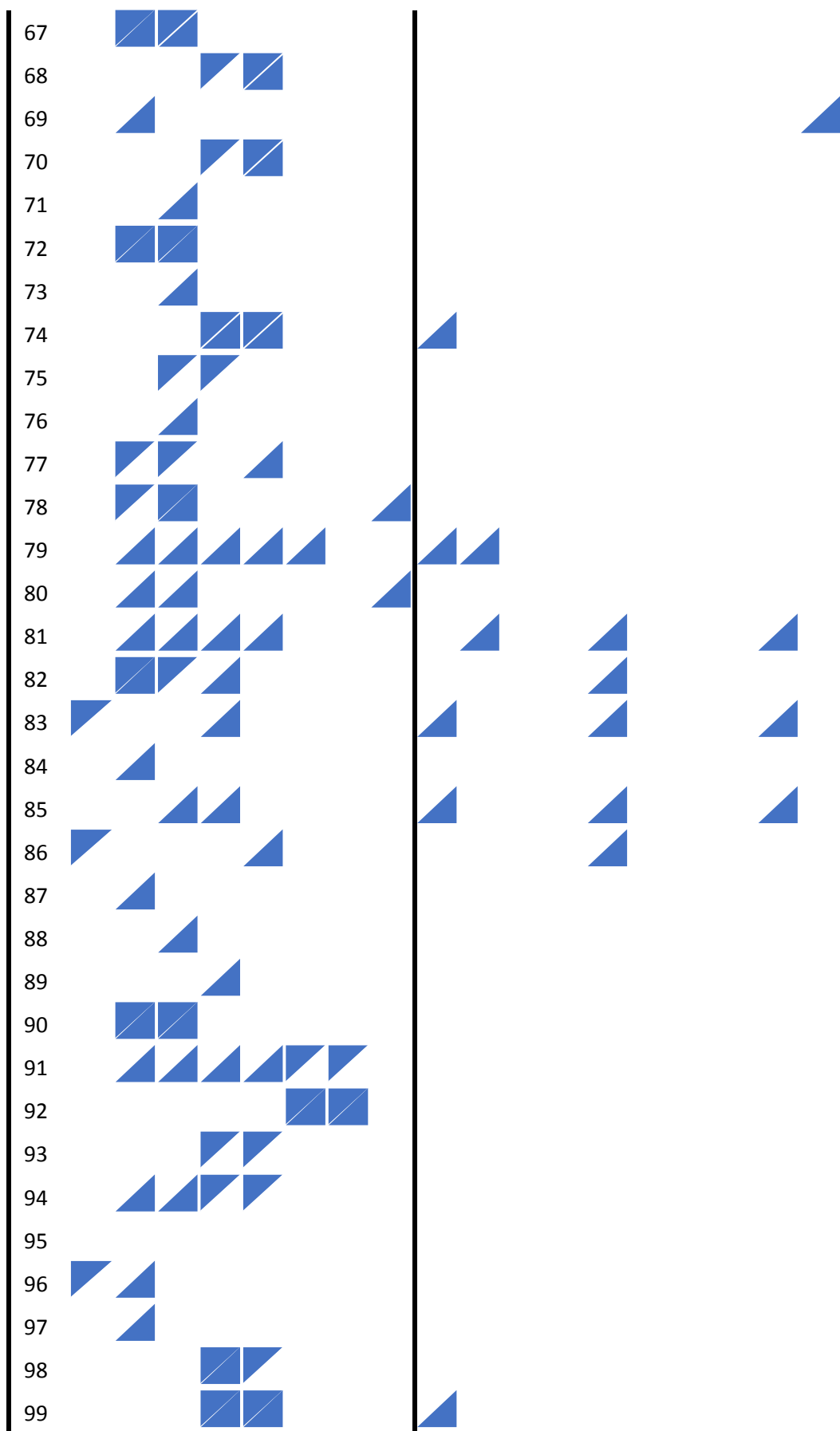

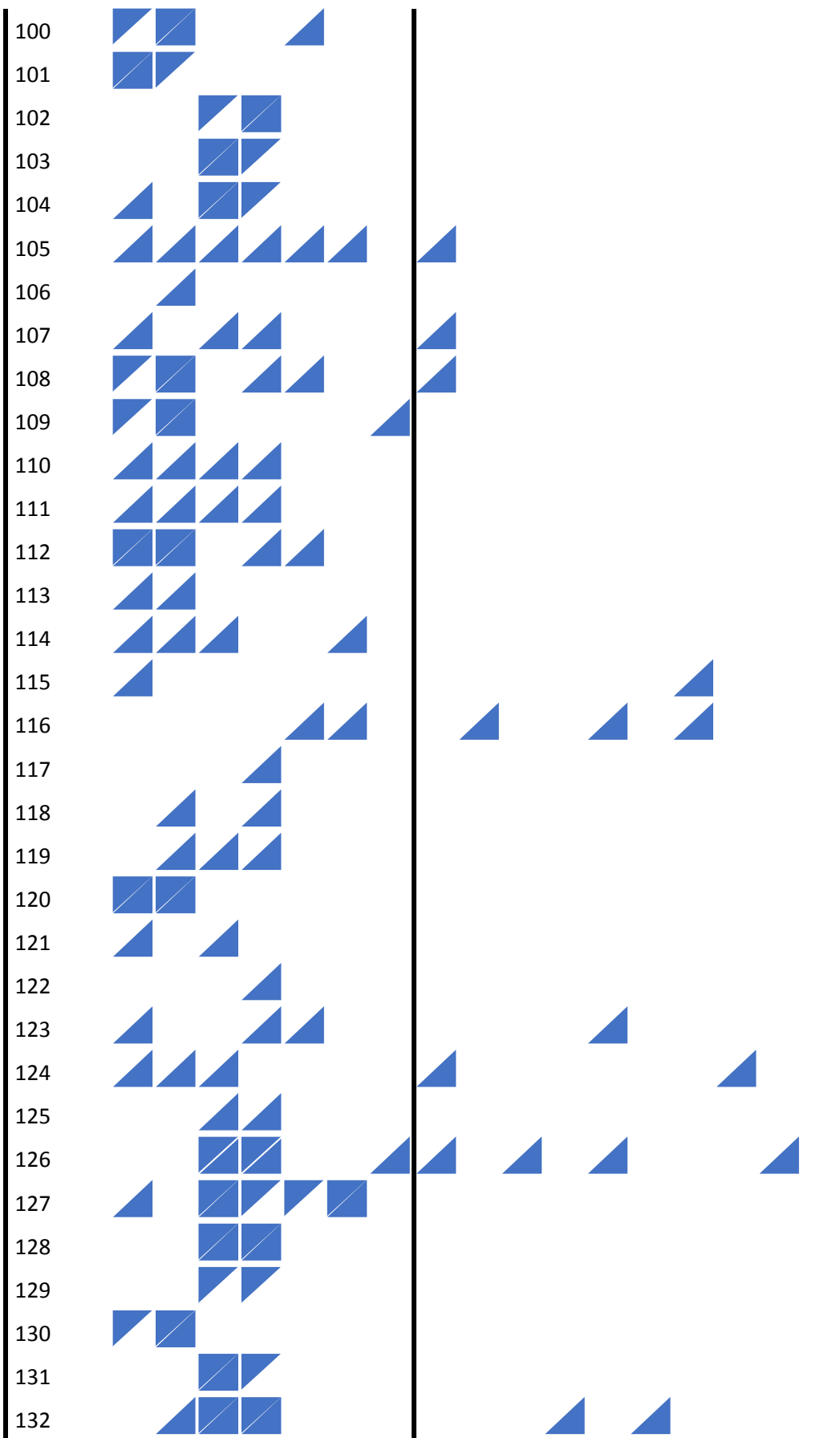

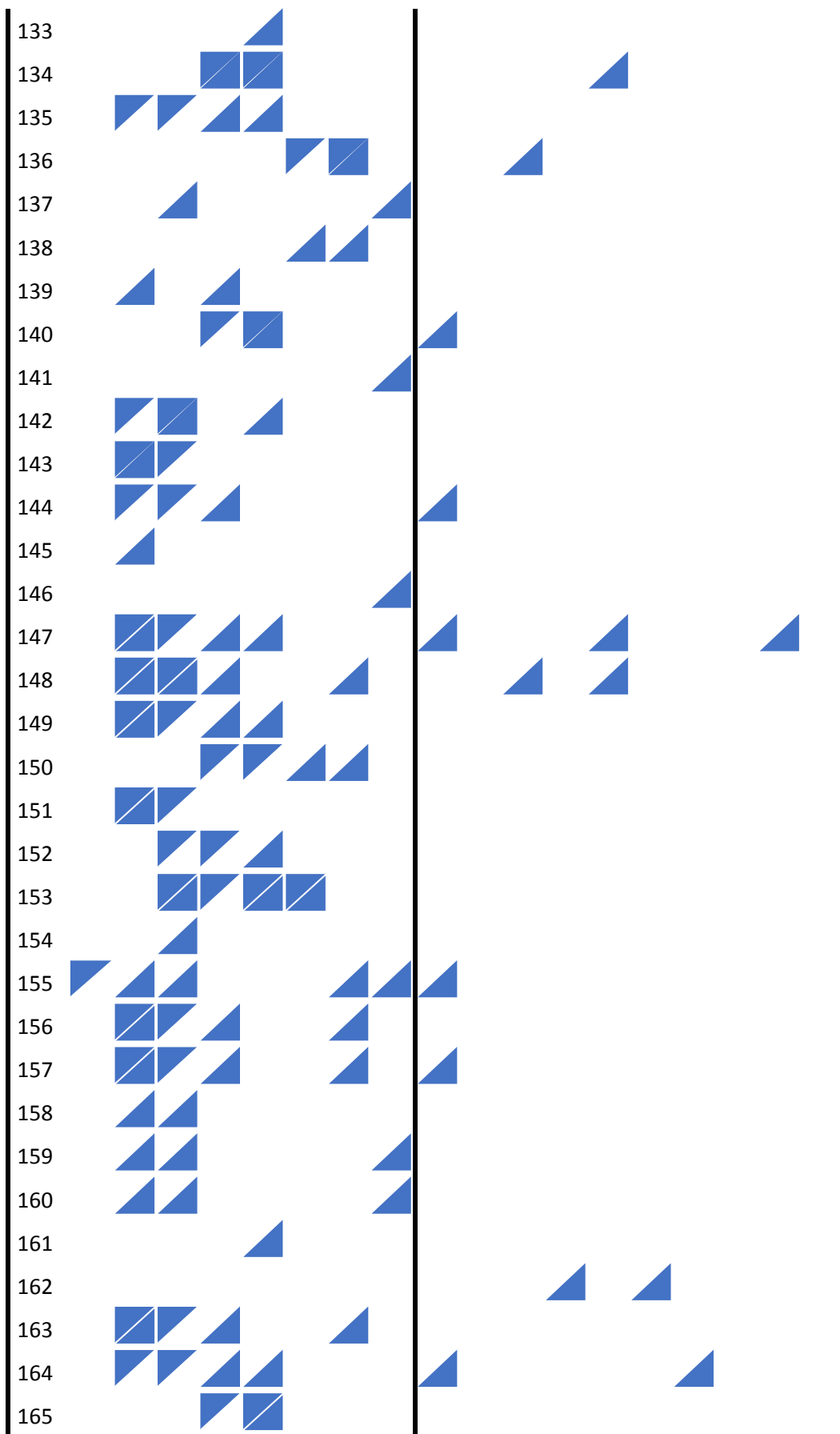

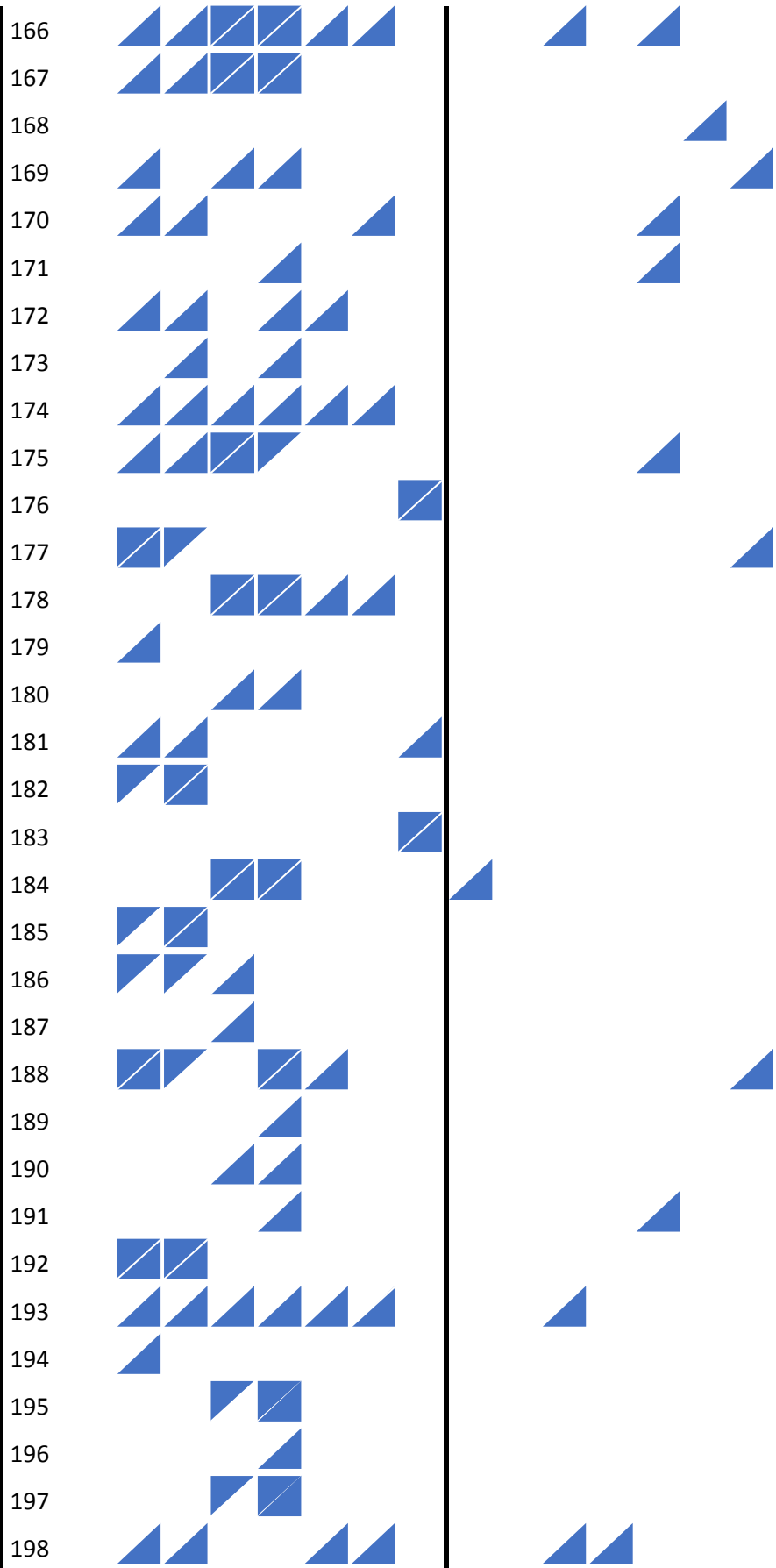

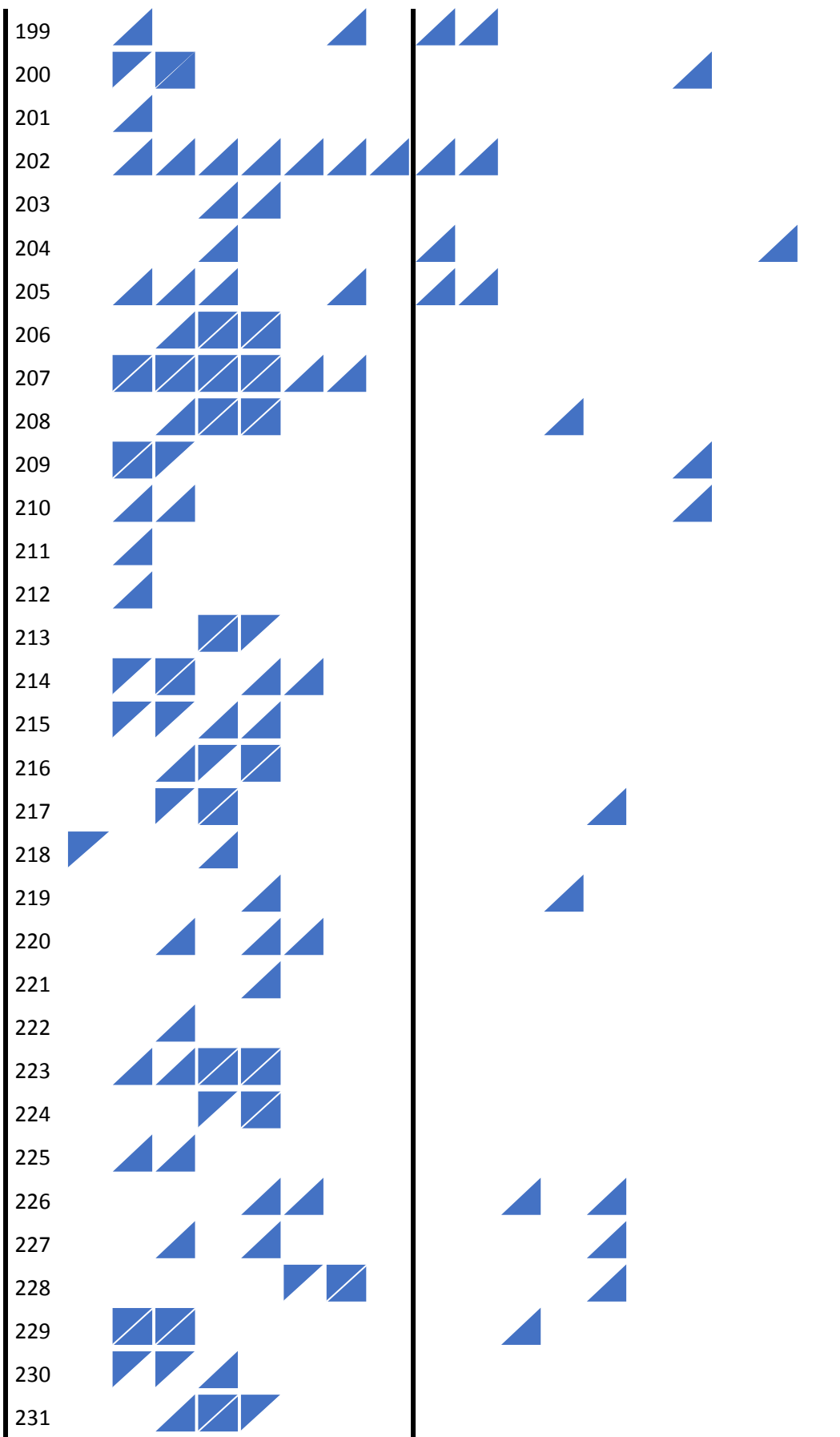

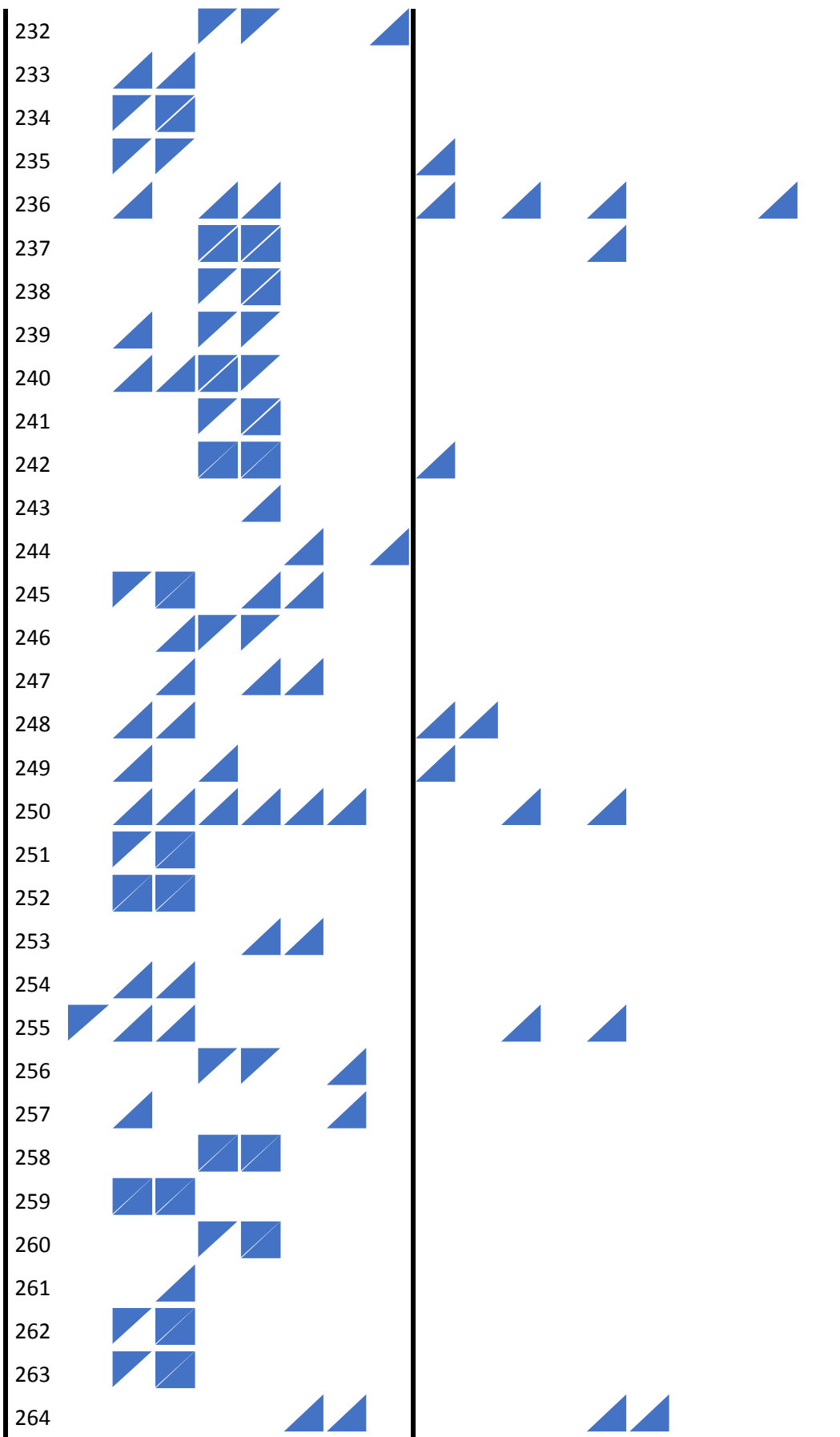

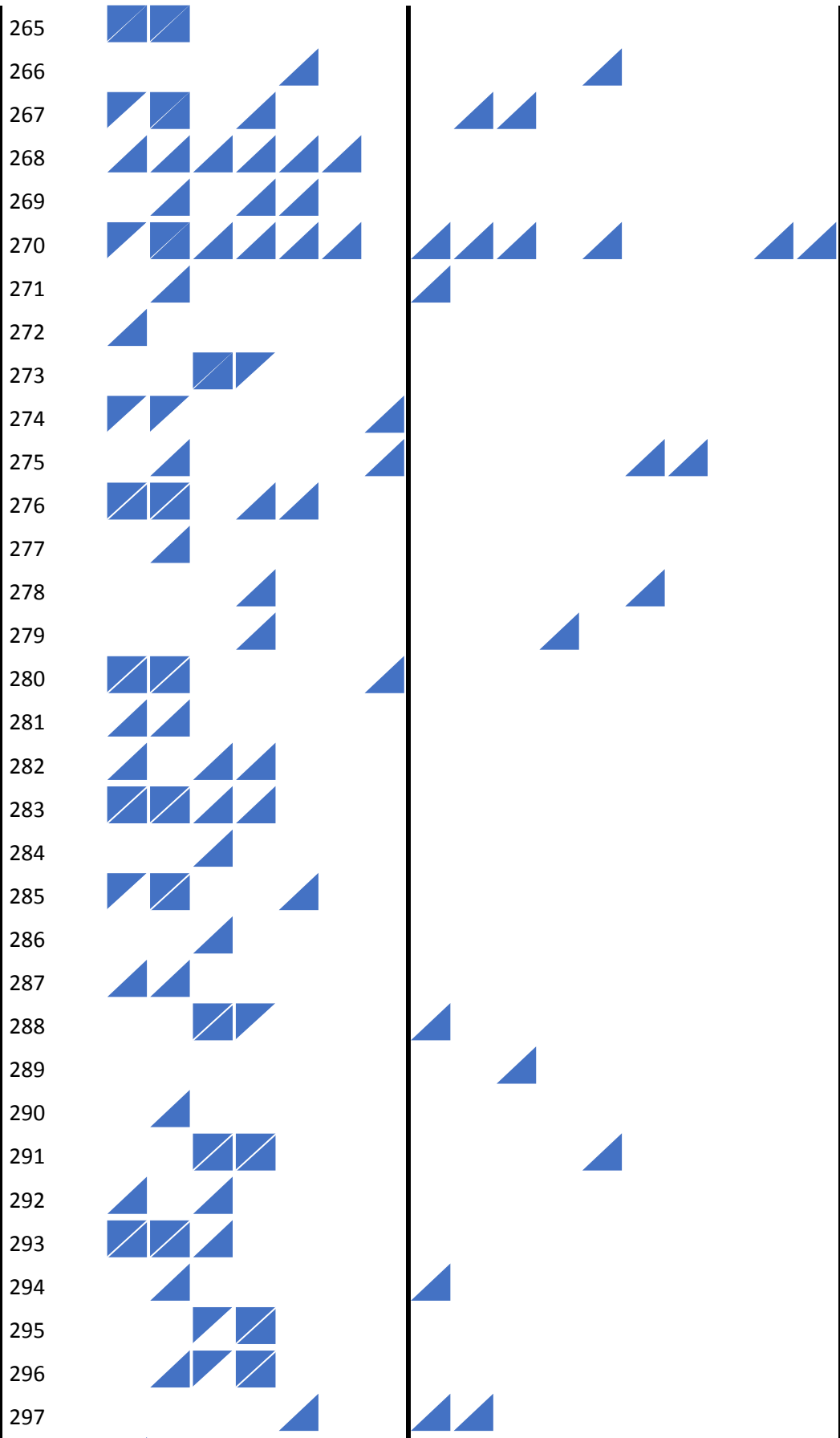

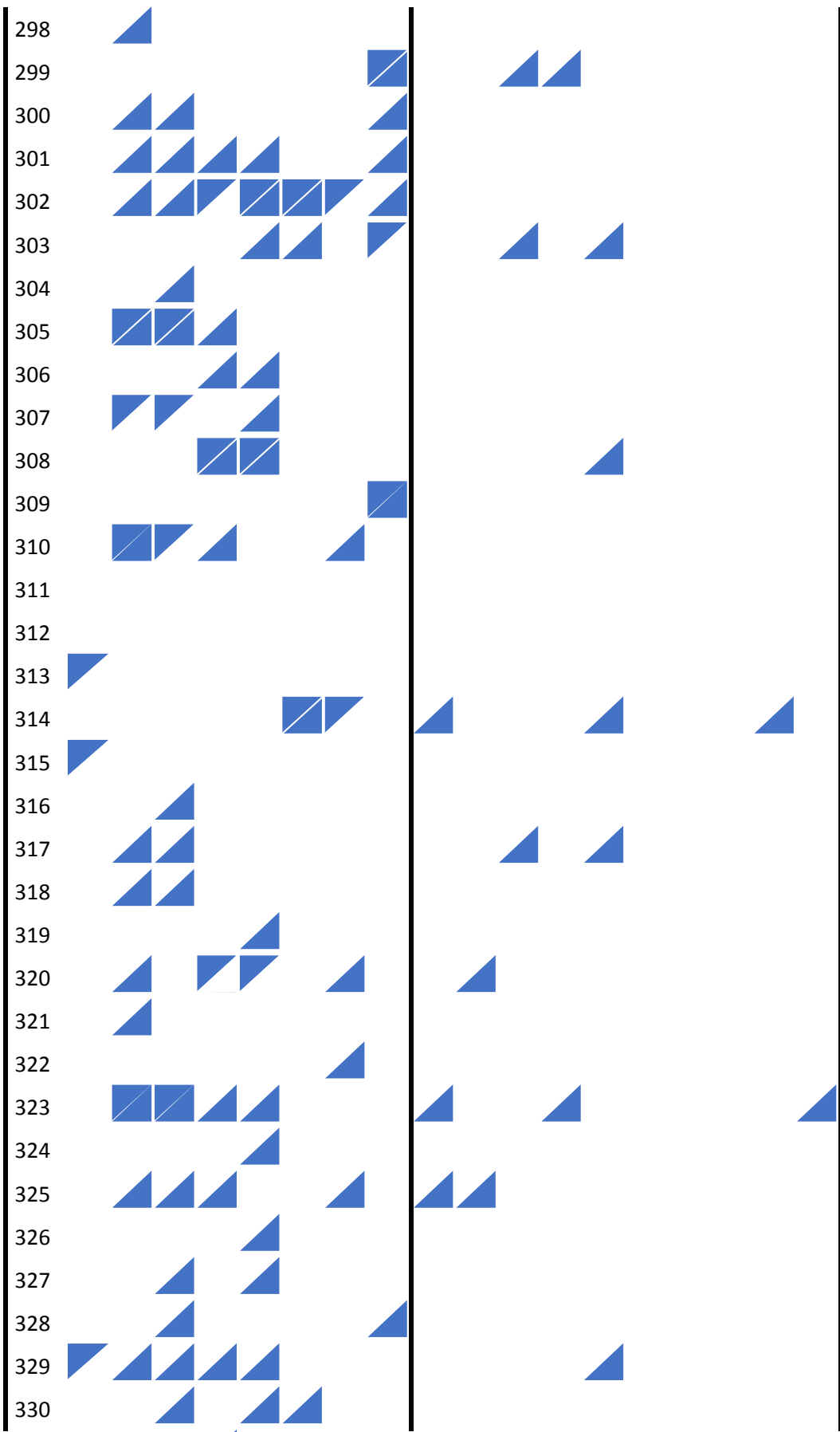



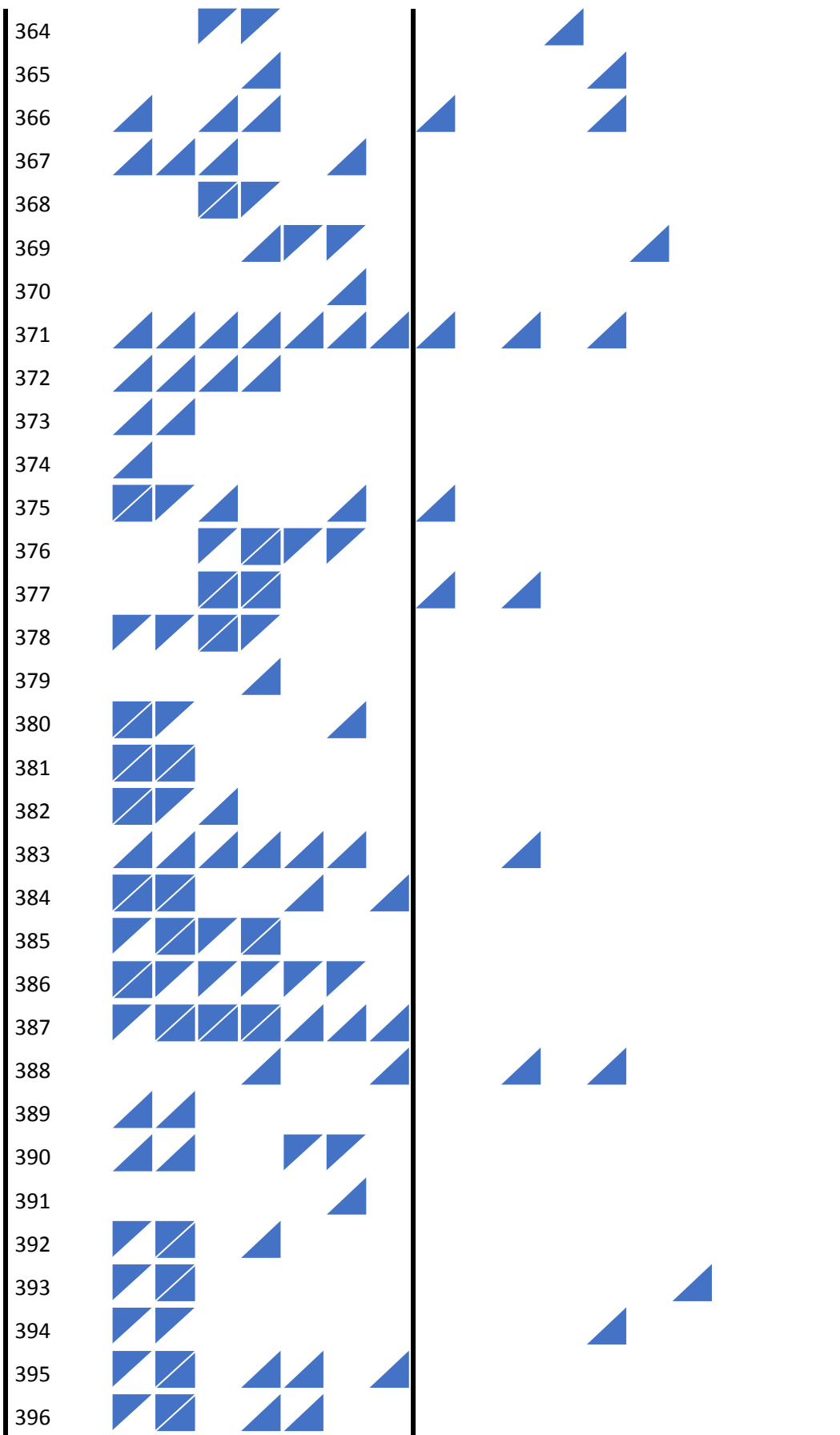

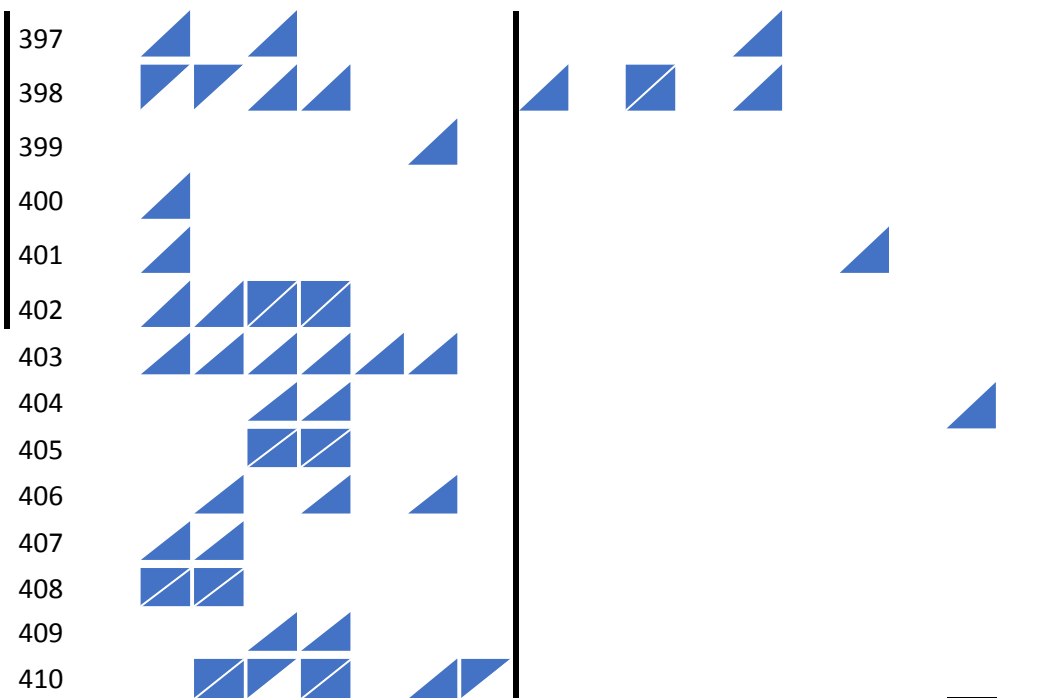

Supplement: S1 Fig — Each line are sites of pain for the same patient. Column numbers refer to regions of the chest displayed in Fig 1. Blue-colored upper triangles for each location of pain are data from the patient’s EHR. Blue-colored lower triangles for each location of pain are data from the patient’s CHT interview. Empty upper triangles indicate that the region was not mentioned as affected by pain in EHR data. Empty lower triangles indicate that the region was not selected by the patient interacting with the image in S1 Fig. Absence of EHR data for location of chest pain indicates that the EHR did not record a specific site of chest pain. Blue-colored upper triangles in Column 0 reflect that the description of chest pain in EHR narratives was too imprecise to be associated with a specific anatomic region of the chest. Projected areas of pain on each line are data for the same patient. (PDF) [file pone.0257677.s001.pdf]

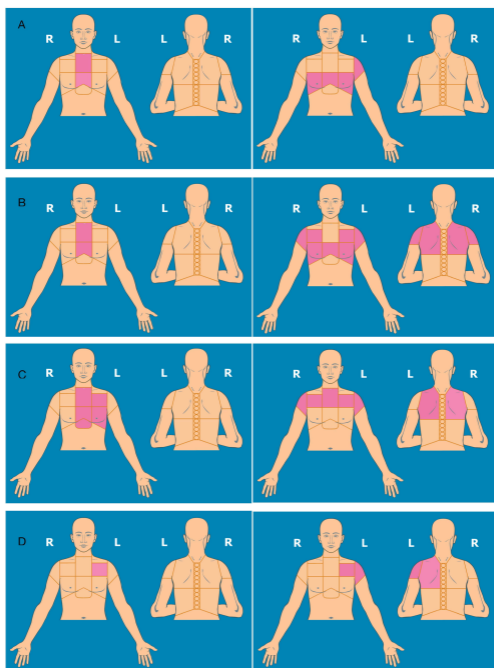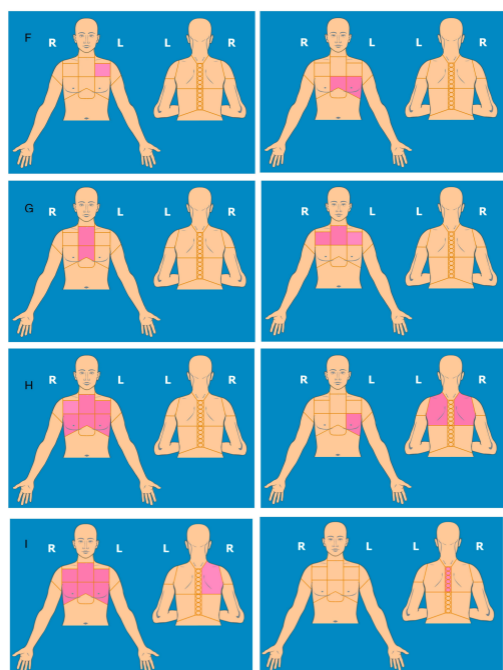

Supplement: S2 Fig — Regions of primary pain projected onto an image of the chest for selected patients recorded in EHR data (left hand panels) and CHT data (right hand panels). (PDF) [file pone.0257677.s002.pdf]
