# Supplementary material for: Computerized history-taking improves data quality for clinical decision-making—Comparison of EHR and computer-acquired history data in patients with chest pain
Source: PLoS One. 2021 Sep 27;16(9):e0257677. doi: 10.1371/journal.pone.0257677 (PMC8476015; doi:10.1371/journal.pone.0257677)
Supplement: Attachment — Submitted filename: Response to reviewers.docx [file pone.0257677.s004.docx]

Replies to reviewers' objections.

Reviewer #1: In my first review of this paper the main issue I took was with a broad over-reaching conclusion based off of likely clinically irrelevant differences between CHT and EMR data. The authors have pared down their claims to a level that fits within the scope of their paper.

However there are still several mentions such as in the abstract, “CHT out-performed history taking by physicians” yet there is still no clinically relevant indication of this.

**Reply**: We removed the phrase and the complete sentence in which it appeared

----------------

While I understand the authors opinion that CHT was created by experts and is likely a better representation of an accurate chest pain history, there is no indication that clinical outcomes are at all effected by this. The authors should carefully re-read their manuscript to eliminate any indication that CHT outperforms EMR history in any manor that would improve clinical outcomes.

**Reply**: There is no mention in the manuscript that CHT will improve outcomes.

---------------
Overall, this version of the manuscript is much stronger. I think that while adding some relevant findings, the results section remains too long and verbose. Much of the “results” section should be moved to discussion instead.

**Reply**: Results has no text not immediately relevant to the primary data. We see no material in Results that fit better in Discussion.

**______________________________________________________________________________**

Reviewer #2: (No Response)

**______________________________________________________________________________**

Reviewer #3: The study authors have made several edits relating to the concerns from the first submission, however the manuscript as it requires further editing or rewriting to be at an acceptable level for publication.

Abstract – lacks objective (statistical) data to support its conclusions

**Reply**: This item is addressed below.

-----------------
Introduction – authors should remove their conclusions from the introduction – specifically “Our data shows that CHT records contain more complete and more accurate representations of patients' perceptions of their symptoms than patients' corresponding EHRs.” Please refer to the STROBE checklist if there is confusion on this point.

**Reply**: We removed this sentence from the Introduction.

Methods – Gender and age data should be reported in the results section. Authors need to include a description of their statistical methods.

**Reply**: Instructions indicated that this data was to be included in Methods.

-----------------
Results – 1. The data on location of primary pain, location of radiated pain, setting of pain onset, and frequency/duration of pain, and data for associated symptoms, use of nitroglycerin, and dimensionality is very difficult to interpret.

The descriptions are very long and would be best presented in table format, with limited narrative.

**Reply**: At the reviewer's suggestion, we added a table (Table 2 in the revised ms) for the data on sites of primary pain. We added a table (Table 4) for data for onset of pain during physical activity or emotional upset. (Data for other variables already is in Tables.)

-------------

Also, many values are reported as integers, where percentages would be better used.

**Reply**: The differences between findings in EHR and CHT data are large for the absolute findings for different variables. Absolute values for what was found enables the reader easily to replicate the statistical test data should they want to do so. We note too that there is more than 1 way to calculate per cent of some number of records because of the large numbers of missing data elements in EHRs. The reader easily can calculate any per cent value that might interest them.

---------------

Finally, only raw data is presented; testing for statistical significance in the different outcomes between the EHR and CHT data sets would greatly strengthen the results.

**Reply**: We have added statistical analyses for all variables. We have added a brief section in Methods describing how statistical significance was determined.

----------------

Discussion - The authors should situate their findings in the context of prior research comparing EHRs and CHTs (ie. this is the first study, or consistent with prior studies, or contrary to prior studies) .

**Reply**: We have added a sentence that contrasts the current work with prior work on this subject.
